# Reinnervation of denervated muscle by implantation of nerve‐muscle‐endplate band graft to the native motor zone of the target muscle

**DOI:** 10.1002/brb3.668

**Published:** 2017-05-03

**Authors:** Liancai Mu, Stanislaw Sobotka, Jingming Chen, Themba Nyirenda

**Affiliations:** ^1^ Department of ResearchHackensack University Medical CenterHackensackNJUSA; ^2^ Department of NeurosurgeryIcahn School of Medicine at Mount SinaiNew YorkNYUSA

**Keywords:** motor endplate, muscle force measurement, muscle reinnervation, native motor zone, nerve regeneration, nerve‐muscle‐endplate band grafting, peripheral nerve injury

## Abstract

**Introduction:**

Motor endplate reinnervation is critical for restoring motor function of the denervated muscle. We developed a novel surgical technique called nerve‐muscle‐endplate band grafting (NMEG) for muscle reinnervation.

**Methods:**

Experimentally denervated sternomastoid muscle in the rat was reinnervated by transferring a NMEG from the ipsilateral sternohyoid muscle to the native motor zone (NMZ) of the target muscle. A NMEG pedicle contained a block of muscle (~ 6 × 6 × 3 mm), a nerve branch with axon terminals, and a motor endplate band with numerous neuromuscular junctions. At 3 months after surgery, maximal tetanic muscle force measurement, muscle mass and myofiber morphology, motoneurons, regenerated axons, and axon‐endplate connections of the muscles were analyzed.

**Results:**

The mean force of the reinnervated muscles was 82% of the contralateral controls. The average weight of the treated muscles was 89% of the controls. The reinnervated muscles exhibited extensive axonal regeneration. Specifically, the mean count of the regenerated axons in the reinnervated muscles reached up to 76.8% of the controls. The majority (80%) of the denervated endplates in the target muscle regained motor innervation.

**Conclusions:**

The NMZ of the denervated muscle is an ideal site for NMEG implantation and for the development of new microsurgical and therapeutic strategies to achieve sufficient axonal regeneration, rapid endplate reinnervation, and optimal functional recovery. NMEG‐NMZ technique may become a useful tool in the treatment of muscle paralysis caused by peripheral nerve injuries in certain clinical situations.

## 1 Introduction

Traumatic peripheral nerve injuries (PNIs) to the head/neck and extremity are a significant cause of morbidity and disability in both military and civil circumstances today (Brininger, Antczak, & Breland, 2008; Eser, Aktekin, Bodur, & Atan, 2009; Kretschmer, Antoniadis, Braun, Rath, & Richter, 2001; Noble, Munro, Prasad, & Midha, 1998). There are 200,000 and 300,000 cases with PNIs in the United States and Europe, respectively, each year (Ichihara, Inada, & Nakamura, 2008; Wiberg & Terenghi, 2003). Approximately, 100,000 patients undergo peripheral nerve surgery in the United States and Europe annually (Kelsey, Praemer, Nelson, Felberg, & Rice, 1997). In the past decades, multiple techniques such as nerve end‐to‐end anastomosis (EEA), end‐to‐side neurorrhaphy, nerve grafting, nerve transfer, muscular neurotization, tubulization techniques, and many others have been developed for the reinnervation of denervated muscles (McAllister, Gilbert, Calder, & Smith, 1996; de Medinaceli, Prayon, & Merle, 1993; Siemionow & Brzezicki, 2009). However, the currently available methods result in poor muscle reinnervation and functional recovery. For example, EEA is commonly used when the two stumps of an injured nerve can be approximated without tension. Unfortunately, only about 50% of patients regain useful function (Lee & Wolfe, 2000; Wong & Crumley, 1995). Factors behind poor functional recovery include tension of the anastomosis, neuroma formation, scaring, and loss of the nerve fiber population (Green, Berke, & Graves, 1991). Studies have shown that in EEA, fewer nerve fibers could pass through the coaptation site and reach the target muscle (Myckatyn & Mackinnon, 2004). When a tension‐free EEA is not technically possible, end‐to‐side neurorrhaphy is an alternative to repair an injured nerve when the proximal nerve stump is unavailable (Al‐Qattan, 2001). The distal stump of an injured nerve is sutured to the side of an intact donor nerve. However, this procedure induces less axon regeneration and functional recovery compared to EEA (De Sa, Mazzer, Barbieri, & Barriera, 2004; Sanapanich, Morrison, & Messina, 2002).

A significant nerve defect is a common clinical situation. At present, autologous nerve grafts, nerve transfers, and tubulization techniques with natural or artificial conduits are used for nerve‐gap repair. Unfortunately, nerve grafting has been associated with poor functional outcomes when there is a long distance from the level of the injury to the target muscle. The recovery rate of motor function after autogenous nerve grafting is <40% (Moneim & Omer, 1998). *Tubulization* techniques are feasible only in short nerve gaps. If the gap exceeds 1.0–1.5 cm, regeneration is poor. Longer defects (4.0–6.0 cm) result in useful reinnervation in only 13% of cases with reconstruction of upper extremity PNIs with conduits (Merle, Dellon, Campbell, & Chang, 1989; Stanec & Stanec, 1998). For the PNIs in which the proximal nerve stump is unavailable, nerve transfer is an option. Nerve transfer is the surgical coaptation of a healthy nerve donor to a denervated nerve. A nerve branch that innervates expendable muscles can be used to repair a functionally more important distal stump of an injured nerve. Many nerve‐to‐nerve transfers have been employed to repair the injured nerves in the hand and upper extremity with mixed results (Lee & Wolfe, 2012). In some cases, nerve repair or nerve‐grafting procedures are inapplicable because of the lack of a nerve stump. In this situation, direct nerve implantation or muscular neurotization may be used (Brunelli & Brunelli, 1980, 1993). In neurotization, the proximal stump of the original nerve or a healthy but less valuable foreign motor nerve is implanted into the target muscle to reinnervate a more important motor territory that has lost its innervation through irreparable damage to its nerve.

More recently, we have developed a new reinnervation technique called “nerve‐muscle‐endplate band grafting (NMEG)” (Mu, Sobotka, & Su, 2011). The development of the NMEG procedure for muscle reinnervation is based on the idea that a paralyzed muscle could be reinnervated by transferring a NMEG from a neighboring donor muscle. A healthy endplate band with a nerve branch and terminals that innervates an expendable muscle can be transplanted to a more functionally important denervated muscle for restoring its motor function. Over the past years, we have studied the NMEG technique by determining innervation patterns (Mu et al., 2011; Zhang, Mu, Su, & Sobotka, 2011) and contractile properties of the rat recipient and donor muscles (Sobotka & Mu, 2010), and conducted surgical feasibility studies and a series of reinnervation experiments using the NMEG technique and conventional EEA in a rat model (Mu et al., 2011; Sobotka & Mu, 2011, 2013a,b, 2015). Several lines of evidence from a number of quantitative analyses demonstrated that the NMEG procedure results in encouraging functional recovery (67% of the control) (Mu et al., 2011). However, entire muscle reinnervation and complete functional recovery was not achieved. We found that approximately one‐third of the distal myofibers in the target muscle were not reinnervated 3 months after surgery (Mu et al., 2011; Sobotka & Mu, 2015). These findings suggest that partial muscle reinnervation accounts for incomplete functional recovery. We assumed that implantation site of the NMEG would be a critical factor influencing outcomes. In our previous studies, a NMEG was implanted into an aneural region in the recipient muscle. In this case, regenerating axons from the NMEG pedicle may need more time to reach the most distal muscle fibers and form new motor endplates (MEPs).

The purpose of this study was to test our hypothesis that optimal outcomes may be achieved by implanting the NMEG into the NMZ in the target muscle, as such a procedure (NMEG‐NMZ) could reduce nerve regeneration distances and facilitate rapid MEP reinnervation. At 3 months after surgery, maximal tetanic force measurement, muscle mass and myofiber morphology, motoneurons, regenerated axons, and axon‐endplate connections of the reinnervated muscles were analyzed and compared with those of the contralateral controls.

## 2 Materials and Methods

### 2.1 Animals

Thirty‐two adult female Sprague‐Dawley rats (Taconic Laboratories, Cranbury, NJ, USA), weighing 200–250 g, were used. The animal experiments were reviewed and approved by the Institutional Animal Care and Use Committee. All animals were handled in accordance with the Guide for Care and Use of Laboratory Animals published by the US National Institutes of Health (NIH publication no. 85–23, revised 1996). The animals were kept in a 22°C environment in a 12:12‐hour light–dark cycle, with food and water ad libitum. The rats were individually housed in standard cages in the state‐of‐the‐art animal housing facilities of Hackensack University Medical Center.

### 2.2 Surgical procedures

Thirty‐two rats were randomly distributed into reinnervation (*n *= 15) and denervation (*n *= 17) groups. In this study, SH and SM muscles were chosen to serve as a donor and recipient, respectively, because they were used in our previous studies (Mu et al., 2011; Sobotka & Mu, 2010, 2013b, 2015). On the day of the experiment, the rats were anesthetized via an intraperitoneal injection of a ketamine (80 mg/kg) and xylazine (5 mg/kg) mixture. With the rat supine, a midline cervical incision was made extending from the hyoid bone to the sternum to expose the right SM and SH muscles and their innervating nerves.

All the rats in both groups underwent denervation of the right SM muscle. With the aid of an Olympus SZX12 Stereo zoom surgical microscope (Olympus America Inc, Center Valley, PA, USA), the right SM muscle was denervated by resecting a 5‐mm segment of its innervating nerve and the cut ends of the nerve were then coagulated with a bipolar cautery to prevent nerve regeneration.

**Figure 1:**
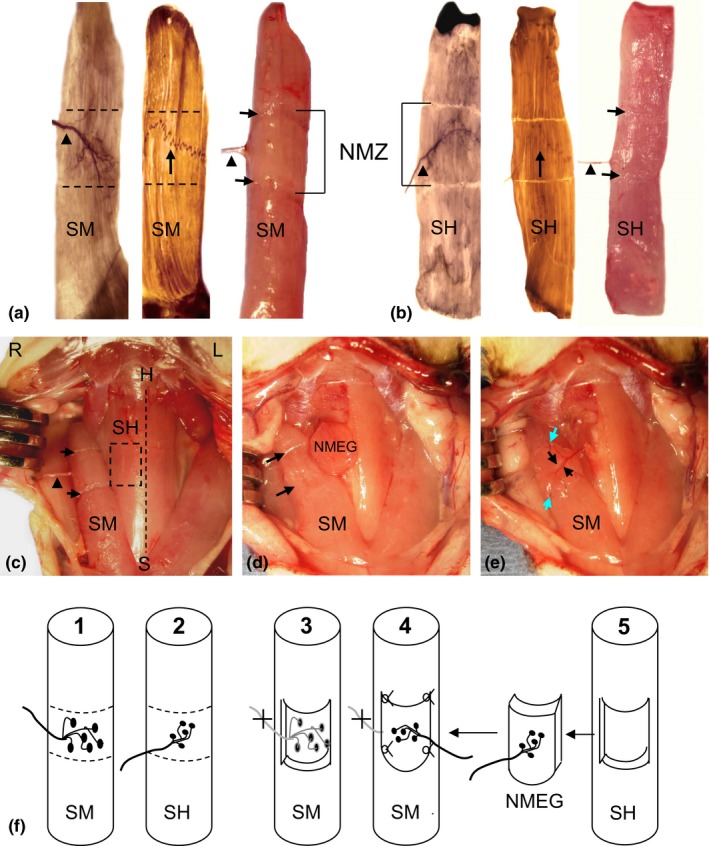
Images showing the locations of native motor zones (NMZ) of the recipient sternomastoid (SM) and donor sternohyoid (SH) muscles of the rat and surgical procedures for NMEG‐NMZ transplantation. (a) Stained and fresh right SM showing the NMZ of the muscle (outlined region). SM nerve (arrowhead) and its intramuscular nerve terminals were mapped out with Shiner's stain (left image), whereas the motor endplate (MEP) band (arrow in the middle image) was visualized with whole mount acetylcholinesterase (AChE) staining. A fresh SM (right image) illustrated the NMZ outlined during surgery (between fiber cuts on the surface of the muscle as indicated by arrows and bracket). (b) Stained and fresh right SH showing that the NMZ of the SH is also located in the middle of the muscle. (c) A photograph from a rat during surgery, showing surgically outlined NMZ in the recipient SM (between fiber cuts as indicated by arrows) and in the donor SH (boxed region). The arrowhead indicates the SM nerve. The dashed line indicates the midline between both SH muscles. H = hyoid bone; L = left; R = right; S = sternum. (d) A NMEG pedicle was harvested from the NMZ of the right SH and a muscular defect (between arrows) was made on the SM muscle. (e) The NMEG was implanted into the recipient bed and sutured (green arrows). The SH nerve branch (large black arrow) and its companying blood vessels (small black arrow) can be seen on the implanted NMEG. (f) Schematic illustrations summarize the surgical procedures by localizing the NMZs of the SM (F1) and SH (F2), denervating the SM and creating a muscular defect in the NMZ of the muscle (F3), and suturing the NMEG pedicle (F4) harvested from the SH (F5)

Immediately after muscle denervation, the rats in reinnervation group were subjected to NMEG‐NMZ transplantation. First, the NMZs in the right SM and SH muscles were outlined according to the motor point (the entry point of the motor branch into the muscle) (Figure 1a and b). The NMZ of either SM or SH in the rat is located in the middle segment of the muscle that contains intramuscular nerve terminals and a MEP band as demonstrated by our previous studies (Mu et al., 2011; Zhang et al., 2011). Second, a muscular defect (recipient bed) with the same dimensions as the NMEG pedicle was made in the NMZ of the right denervated SM muscle (Figure 1c and d). Third, a NMEG pedicle was harvested from the NMZ of the right SH donor muscle as described in our previous publication (Mu et al., 2011). Briefly, the SH nerve branch was identified on the lateral margin of the NMZ of the muscle. A NMEG pedicle containing a block of muscle (~ 6 × 6 × 3 mm), axon terminals, and a MEP band with numerous neuromuscular junctions was outlined and harvested from the SH muscle in continuity with its pedicle of motor nerve branch and feeding vessels (Figure 1e). A functioning NMEG was confirmed by observing its twitch contractions on nerve stimulation. Fourth, the superficial muscle fibers on the ventral aspect of the NMEG pedicle were removed to create a denuded surface for better neural regeneration. Finally, the well‐prepared NMEG was embedded in the SM muscle defect and sutured with four to six 10‐0 nylon microsutures (Figure 1e). Thus, the denervated SM muscle was reinnervated with NMEG‐NMZ technique. Figure 1f summarizes the surgical procedures for NMEG‐NMZ implantation. After surgery, the wound was closed in layers with interrupted simple sutures of 4‐0 prolene.

### 2.3 Postoperative evaluations

At the end of the 3‐month recovery period, outcomes of the NMEG‐NMZ technique was evaluated functionally and histochemically. The removed SM muscles from the rats in the reinnervation and denervation groups were analyzed using histological and immunohistochemical methods.

#### 2.3.1 Determining the degree of functional recovery

Maximal tetanic force measurement was used to evaluate the functionality of the NMEG‐NMZ reinnervated muscles. The force was measured on the experimental and the contralateral control side and expressed in percentage of the control side. Muscle force measurement is a useful approach to assess the mechanical function and contractile properties of a muscle (van der Meulen, Urbanchek, Cederna, Eguchi, & Kuzon, 2003; Yoshimura et al., 2002). Our studies (Mu et al., 2011; Sobotka & Mu, 2011, 2013a,b, 2015) and others’ (Goding, Cummings, & Bright, 1989; Marie et al., 1999; Meikle, Trachy, & Cummings, 1987) used force measurement to detect the degree of functional recovery of the reinnervated cervical strap muscles. The details regarding the force measurement of the rat SM muscle have been given in our publications (Mu et al., 2011; Sobotka & Mu, 2010, 2011, 2013a,b, 2015). Briefly, the right reinnervated and the left control SM muscles in each animal were exposed and dissected free from the surrounding tissues. The rostral tendon of each muscle was severed close to the insertion, tied with a 2–0 suture, and attached to a servomotor lever arm (model 305B Dual‐Mode Lever Arm System; Aurora Scientific Inc, Aurora, Ontario, Canada). Muscle force of the right reinnervated SM was measured by stimulating the SH nerve branch supplying the NMEG, whereas that of the left control muscle was measured by stimulating the intact SM nerve. Each of the nerves was identified, isolated, and draped over a bipolar stimulating electrode for nerve stimulation. Trains of biphasic rectangular pulses of different current were delivered to the stimulated nerve using our stimulation and recording system that is built based on a multifunction National Instruments Acquisition Board (National Instruments Corp, Austin, TX, USA) and is controlled by user‐written LabVIEW software (National Instruments Corp).

Isometric contraction of the SM muscle was produced with 200‐millisecond trains of biphasic rectangular pulses. The duration of each phase of stimulation pulse was set at 0.2 milliseconds and the train frequency was set at 200 pulses per second. The stimulation current was gradually increased until the tetanic force reached a plateau. A break of at least 1 min was taken between two stimulations. The maximum value of muscle force during the 200‐millisecond contraction was identified, as well as initial passive tension before stimulation. The difference between the maximal active force and the preloaded passive force was used as the muscle force measurement. The force generated by the contraction of the SM muscle was transduced with the servomotor of a 305B lever system and displayed on a computer screen. At the moment of force measurement, the lever arm was stationary, and the muscle was adjusted to the optimal length for the development of maximum force. During the experiment, the animal was placed supine on a heating pad (homoeothermic blanket system; Stoelting, Wood Dale, IL, USA). The core body temperature was monitored with a rectal thermistor and maintained at 36°C. The muscle and nerve examined were bathed regularly with warmed mineral oil throughout the testing to maintain muscle temperature between 35°C and 36°C.

The force data were obtained and processed by an acquisition system built from a multifunction I/O National Instruments Acquisition Board (NI USB 6251; 16 bit, 1.25 Ms/s; National Instruments) connected to a Dell laptop with a custom‐written program using LabVIEW 8.2 software. The system produced stimulation pulses, which, after isolation from the ground through an optical isolation unit (Analog Stimulus Isolator model 2200; AM Systems, Inc, Carlsborg, Washington, DC, USA), were used for the current controlled nerve stimulation. The acquisition system was also used to control muscle length and to collect a muscle force signal from the 305B Dual‐Mode Lever System. Collected data were analyzed offline with DIAdem 11.0 software (National Instruments).

#### 2.3.2 Tracking the origin of the axons in the SM muscles reinnervated with NMEG‐NMZ technique

Immediately after tetanic tension measurements, five experimental animals were subjected to retrograde horseradish peroxidase (HRP) neuronal labeling to track the origin of the axons innervating the reinnervated SM muscles. As the NMEG was harvested from the right SH muscle and transplanted into the denervated right SM muscle, the HRP injected into the right SM should label SH motoneurons if muscle reinnervation was successful. The details regarding HRP labeling have been given in our previous publications (Mu et al., 2011; Zhang et al., 2011). Briefly, 30% HRP (type VI A; Sigma Chemical, St. Louis, MO, USA) solution was injected at two points (5 μl per point) into the MEP band in the right reinnervated SM and the left SH (control) with Hamilton microsyringe. After a survival time of 48 h, the animals were deeply anesthetized with sodium pentobarbital (60 mg/kg intraperitoneally) and perfused transcardially with 200 ml of a fixative solution containing 1% paraformaldehyde, 1.25% glutaraldehyde, and 3% dextrose in 0.1 mol/L phosphate buffer at pH 7.4 and 4°C. After perfusion, the fixed lower medulla oblongata and cervical spinal cord (C1‐C4) were dissected out, postfixed 4 hr at 4°C in the same fixative, and then stored in graded concentrations (10% and 25%) of phosphate‐buffered sucrose solution at pH 7.4 and 4°C for 2 hr and overnight, respectively. The fixed medulla and upper cervical spinal cord were frozen and sectioned at 40 μm on a cryostat in the transverse plane. The sections were reacted for 20 min in the dark with 3,3′,5,5′‐tetramethylbenzidine as the chromogen to develop the HRP reaction product. The processed sections were mounted on gelatin‐coated slides and counterstained with 1% aqueous neutral red (pH 4.8).

The stained serial sections were examined under a Zeiss photomicroscope (Axiophot‐2; Carl Zeiss, Goettingen, Germany) and photographed with a digital camera (Spot‐32; Diagnostic Instruments, Keene, NH, USA) attached to the photomicroscope. The distributions of the SM and SH motoneurons in the medulla oblongata and spinal cord were recorded to see if the right‐treated SM was controlled by SH motoneurons.

#### 2.3.3 Examining muscle mass, implanted NMEG, and myofiber morphology

At the end of the experiments, SM muscles on both sides in each animal were removed, weighed, and photographed. A muscle mass ratio was calculated (muscle mass ratio = weight of reinnervated muscle/weight of contralateral muscle). Five NMEG‐NMZ reinnervated right SM muscles were processed with Sihler's stain, a whole mount nerve mapping technique to see if the NMEG was precisely implanted into the NMZ of the target muscle. The details regarding Sihler's stain have been given in our previous publication (Mu & Sanders, 2010). Briefly, the removed muscles were fixed for 3 weeks in 10% unneutralized formalin; macerated for 2 weeks in 3% aqueous potassium hydroxide (KOH) solution; decalcified for 1 week in Sihler solution I (one volume glacial acetic acid, one volume glycerin, and six volumes 1% w/v aqueous chloral hydrate); stained for 3 weeks in Sihler solution II (one volume stock Ehrlich's hematoxylin, one volume glycerin, and six volumes 1% w/v aqueous chloral hydrate); destained for 3 hr in Sihler solution I; immersed for 1 hr in 0.05% w/v lithium carbonate solution to darken the nerves; cleared for 3 days in 50% v/v aqueous glycerin; and preserved for 4 weeks in 100% glycerin with a few thymol crystals for transparency. After transillumination by a xenon light source (model 610; Karl Storz, Endoscopy‐America, Culver City, CA, USA), the stained muscles were dissected and photographed.

The removed SM muscles (*n *= 30) from 10 rats with NMEG‐NMZ reinnervation and five rats with denervation were sectioned and stained using histological and immunohistochemical techniques. Each SM muscle was divided into three segments: rostral, middle, and caudal. The muscle segments were frozen in melting isopentane cooled with dry ice and cut on a cryostat (Reichert‐Jung 1800; Mannheim, Germany) at –25°C. The rostral and caudal segments were cut transversely and serial cross sections (10 μm) were stained with routine hematoxylin and eosin (H&E) staining to examine the effects of NMEG‐NMZ reinnervation or denervation on the muscle mass and myofiber morphology. In contrast, the middle muscle segment containing the NMZ and/or NMEG was cut sagittally (60 μm) and sections were stained using immunohistochemical methods to detect axons and MEPs as described below.

#### 2.3.4 Documenting nerve regeneration and MEP reinnervation

##### Immunohistochemical staining for neurofilament (NF)

Some sagittal sections were immunostained with a monoclonal antibody against phosphorylated NF (SMI‐31; Covance Research Products, Berkeley, CA, USA) as a marker for all axons as described in our previous publication (Mu et al., 2015). Briefly, the sections were: (1) treated in PBS containing 0.3% Triton and 2% BSA for 30 min; (2) incubated with primary antibody SMI‐31 (dilution 1:800) in PBS containing 0.03% Triton at 4°C overnight; (3) incubated for 2 hr with the biotinylated secondary antibody (anti‐mouse, 1:1000; Vector, Burlingame, CA, USA); (4) treated with avidin‐biotin complex method with a Vectastain ABC kit (1:1000 ABC Elite; Vector); and (5) treated with diaminobenzidine‐nickel as chromogen to visualize peroxidase labeling. Control sections were stained as described except that the incubation with the primary antibody was omitted.

The muscle sections immunostained for NF permit to show how the regenerating axons from the implanted NMEG reinnervate the denervated SM and quantify the density and spatial distribution of the regenerating axons in the target muscle. The extent of axonal regeneration and muscle reinnervation was evaluated by quantifying the NF‐immunoreactive (NF‐ir) axons within the treated muscles as described in our previous publications (Sobotka & Mu, 2013a, 2015). The intramuscular axonal density was assessed by estimating the number of the NF‐ir axons and the area fraction of the axons within a section area (1.0‐mm^2^). For a given muscle, three sections stained for NF were selected at different spatial levels to count NF‐ir axons. For each section, five microscopic fields with NF‐ir axons were identified and photographed in an order from ventral to dorsal aspect of the muscle. Areas with NF‐positive staining were outlined, measured with public domain ImageJ software (v. 1.45s; NIH, Bethesda, Maryland). For each rat, the number and the area fraction of the NF‐ir axons in the operated muscle were compared with those in the contralateral control.

##### Double fluorescence staining

Double fluorescence staining was used to label axons and MEPs as described (Grumbles, Sesodia, Wood, & Thomas, 2009) with some modifications. Briefly, some sagittal muscle sections were: (1) dried and then placed in Zamboni fixative with 5% sucrose for 20 min at 4°C; (2) washed several times with 1.5‐T buffer with 0.05% Tween‐20 and treated with 0.1 mol/L of glycine in 1.5 T buffer for 30 min, and dipped in 100% methanol at −20°C; (3) blocked in 1.5 T buffer containing 4% normal goat serum for 30 min to inhibit nonspecific protein binding; (4) incubated overnight at 4°C with primary antibodies (SMI‐31 to detect neurofilaments and SMI‐81 to label thinner axons; Covance Research Products Inc); (5) washed in 1.5 T buffer with 0.05% Tween‐20 and incubated at room temperature for 2 hr both with a secondary antibody (goat anti‐mouse antibody conjugated to Alexa 488) and with α‐bungarotoxin conjugated with Alexa 596 (Invitrogen Corporation, Carlsbad, CA, USA); and (6) washed in 1.5 T buffer with 0.05% Tween‐20 and coverslipped.

The stained sections were viewed under a Zeiss fluorescence microscope and photographed. SMI‐31 and SMI‐81 detected axons (green), while α‐bungarotoxin labeled postsynaptic acetylcholine receptor (AChR) site in the MEPs (red). For each muscle sample, at least 100 labeled MEPs were randomly selected from three stained sections at different spatial levels through the muscle to determine the percentages of the innervated (visible axon attachment) and noninnervated (no visible axon attachment) MEPs.

### 2.4 Data analysis

Wet muscle weights, force values, the number and area fraction of NF‐ir axons, and innervated and noninnervated MEPs of the SM muscles in each rat were computed. The variables of the reinnervated SM muscles were expressed as the percentages of the values of the contralateral control muscles. All data were reported as mean ± SD. The Student *t‐*test (paired, two tailed) was used to compare differences in the mean muscle force, mean muscle weight, and mean number and area fraction of NF‐ir axons between operated and unoperated SM muscles. A difference was considered statistically significant at *p *<* *0.05.

## 3 Results

### 3.1 Maximal tetanic muscle force

**Figure 2:**
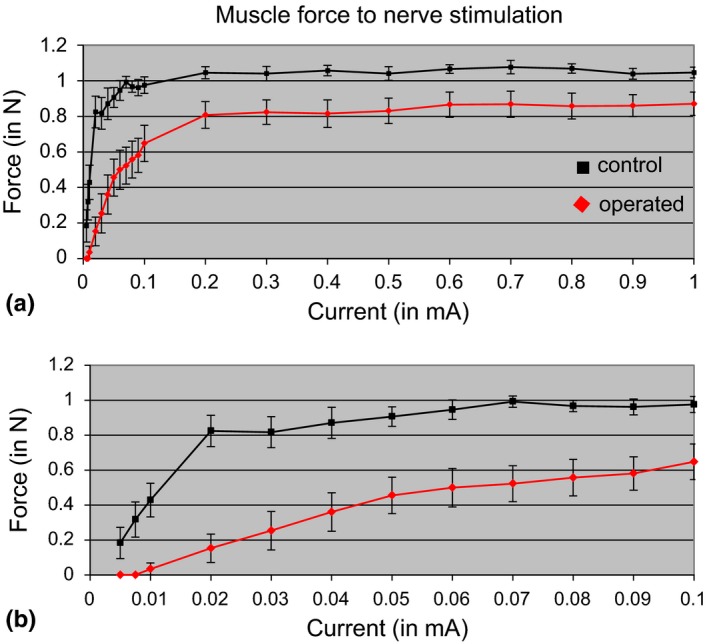
Muscle force as a function of stimulation current in operated and control SM muscles. The passive tension was set at a moderate level (0.08 N). Nerve stimulation was made with a 0.2‐second train of 0.2‐millisecond‐wide biphasic pulses at frequency of 200 Hz. The lower graph (b) shows in expanded scale the early portion of the upper graph (a). Operated SM muscle with implanted NMEG pedicle (shown in red) when compared to control muscle at the opposite side (shown in black) has larger stimulation threshold, reach the level of maximal force with larger stimulation current and has smaller maximal force. Maximal muscle force level was calculated as the average muscle force to stimulation currents from 0.6 to 1 mA. Average maximal muscle force level at the operated side (0.865 N) was 81.6% of muscle force at the control side (1.060 N). Vertical bars represent the standard deviation of the mean

Maximum tetanic tension in the NMEG‐NMZ reinnervated SM and the contralateral control muscles were evaluated at the end of the 3‐month recovery period. The averaged force values for the treated and control muscles are summarized in Figure 2. For each rat, the percentage of functional recovery of the SM muscle reinnervated with NMEG‐NMZ technique was determined as compared with that of the contralateral control muscle. Our previous studies have demonstrated that optimal muscle length could be achieved by stretching the muscle with moderate tension of 0.08 N (Mu et al., 2011; Sobotka & Mu, 2010). Electric stimulation of the nerve branch innervating the NMEG at low intensity (0.02–0.03 mA) produced visible muscle contraction. A lower (0.0075–0.01 mA) stimulation threshold was obtained when the nerve innervating the left intact SM muscle was stimulated. An increase in the stimulation current resulted in an increase in muscle force until it reached horizontal asymptote. During nerve stimulation on the operated side, this saturation level was reached with 0.2 mA. The saturation level was reached at smaller current (0.1 mA) on the control side. The reinnervated SM showed an average maximum tetanic force of 0.87 ± 0.23 N (control, 1.06 ± 0.10 N; *p *<* *0.005). Therefore, the reinnervated SM muscles produced 82% of the maximal tetanic tension of the contralateral control muscles.

### 3.2 HRP‐labeled motoneurons innervating NMEG‐NMZ reinnervated SM muscle

**Figure 3:**
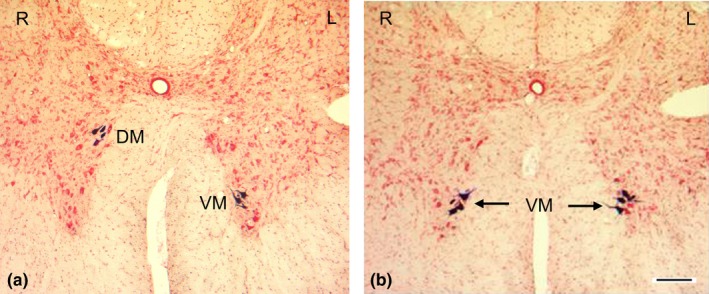
Motoneurons labeled with retrograde horseradish peroxidase (HRP) neuronal tracing in rats. (a) A transverse section from upper cervical spinal cord segment C1 in a normal adult rat, showing different locations of the sternomastoid (SM) and sternohyoid (SH) motoneurons. The HRP solution was injected into the right (R) SM and left (L) SH muscles. Note that in C1, the SH motoneurons were confined to the ventromedial (VM) region, whereas SM motoneurons were located in the dorsomedial (DM) region of the ventral horn of the spinal cord. (b) A transverse section from C1 after HRP injections into the right reinnervated SM muscle and the left intact SH muscle in a rat. Note that the motoneurons controlling the right reinnervated SM were located in the same region as that innervating the SH (VM in C1). Bar = 100 μm

The location of HRP‐labeled motoneurons on the operated side could demonstrate the source of innervation of the NMEG‐NMZ reinnervated muscle. As reported in our previous studies, the SM motoneurons were concentrated in the lower medulla oblongata and spinal ventral grey horn in the C1 to C2 (Mu et al., 2011; Zhang et al., 2011). The SM motoneurons in C1 were concentrated mainly in the dorsomedial region, whereas the SH motoneurons at this level were located in the ventromedial region of the ventral horn (Mu et al., 2011). In this study, HRP injections into the right reinnervated SM muscle labeled motoneurons in the ventromedial region of the ventral horn in the C1 to C2, which were consistent with those labeled by HRP injections into the left intact SH muscle (Figure 3). These findings indicate that the axons innervating the treated SM muscles were derived solely from the implanted SH nerve and controlled by the SH motoneurons. Retrograde HRP labeling also confirmed that the NMEG‐NMZ microsurgery was successfully performed.

### 3.3 Muscle weight, location of the implanted NMEG, and myofiber morphology

**Figure 4:**
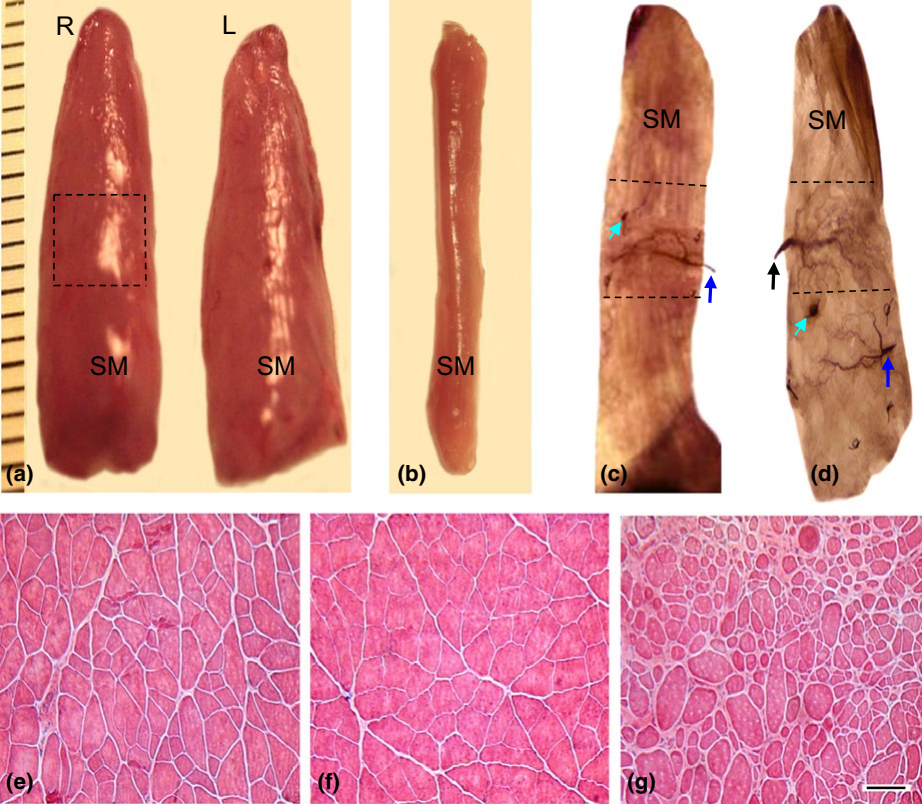
Images from the reinnervated, normal control, and denervated SM muscles showing gross appearance, muscle mass, myofiber morphology, and NMEG implantation site. (a) A pair of SM muscles removed from a rat 3 months after NMEG‐NMZ surgery. Note that the mass of the right (R) reinnervated SM muscle was close to that of the left (L) control muscle. The boxed region in the right SM is the location of the implanted NMEG. (b) A SM muscle denervated by resecting a 5‐mm segment of its innervating nerve for 3 months. Note that the denervated SM showed a more significant loss of muscle mass as compared with the reinnervated and normal SM muscles. (c–d) Sihler's stained SM muscles showing the difference in the NMEG implantation site between NMEG‐NMZ technique (c) and our originally designed NMEG procedure (d). Note that the NMEG pedicle containing a SH nerve branch (blue arrow) and intramuscular nerve terminals was implanted into the NMZ (outlined region in c) of the SM in the middle portion of the muscle for NMEG‐NMZ reinnervation. In contrast, the NMEG pedicle was implanted into the caudal MEP‐free area of the target muscle in our originally designed NMEG procedure (d). Green arrow indicates a microsuture surrounding the implanted NMEG pedicle. The implanted NMEG contained a SH nerve branch (blue arrow) and nerve terminals. Black arrow in d indicates the SM nerve branch. (e–g) Hematoxylin and eosin‐stained transverse sections of the SM muscles. Note that 3 months after surgery, NMEG‐NMZ reinnervated SM (e) exhibited very good preservation of muscle structure and myofiber morphology with less fiber atrophy as compared with the normal (f) and denervated (g) muscles. Bar = 100 μm for e through g

In each rat, gross appearance and size of the operated SM was similar to those of the contralateral control muscle (Figure 4a). The reinnervated SM muscles were greater in size than the SM muscles with complete denervation for 3 months (Figure 4b). The mean value and standard deviation of the wet weight was 0.332 ± 0.047 g for the reinnervated SM muscles, whereas 0.374 ± 0.044 g for the contralateral control muscles (Table 1). The differences in muscle weights of the reinnervated and control muscles were relatively small but consistent across all animals and therefore statistically significant (*p *<* *0.0001). The reinnervated SM muscles weighed 89% of the weight of contralateral control muscles (Table 1). The mean percent of wet weight (89% of the control) of NMEG‐NMZ reinnervated SM muscles was much higher than that of the denervated SM muscles (44% of the control; *p *<* *0.0001).

**Table 1:** Wet muscle weight measurement for the right reinnervated and left control sternomastoid (SM) muscles in rats (*n *= 15)

Animal no.	Body weight, g	Right SM, g	Left SM, g	Ratio R/L
1	333	0.309	0.333	0.928
2	328	0.332	0.397	0.836
3	319	0.318	0.357	0.891
4	285	0.287	0.328	0.875
5	386	0.245	0.316	0.775
6	373	0.373	0.407	0.916
7	281	0.320	0.343	0.933
8	330	0.396	0.437	0.906
9	329	0.305	0.327	0.933
10	355	0.335	0.358	0.936
11	380	0.341	0.392	0.870
12	304	0.326	0.338	0.964
13	358	0.433	0.462	0.937
14	315	0.373	0.410	0.910
15	328	0.290	0.398	0.729
Average	333.6	0.332	0.374	0.889
STDEV	31.8	0.047	0.044	0.065

L, left; R, right.

The location of the implanted NMEG within the right SM muscle was determined. Sihler's stain showed that microsurgery for NMEG‐NMZ reinnervation was successfully performed as indicated by precise implantation of the NMEG into the NMZ of the target muscle (Figure 4c). Each of the implanted NMEG contained a SH nerve branch and numerous nerve terminals, indicating that the treated SM was reinnervated by the SH nerve. In our original NMEG procedure, however, the NMEG pedicle obtained from the ipsilateral SH muscle was implanted into the caudal endplate‐free area in the SM muscle (Figure 4d).

Hematoxylin and eosin‐stained transverse muscle sections showed that the NMEG‐NMZ reinnervated SM muscles exhibited very good preservation of muscle structure and myofiber morphology with less fiber atrophy (Figure 4e) as compared with the contralateral control (Figure 4f) and denervated (Figure 4g) muscles.

### 3.4 Axonal regeneration and MEP reinnervation

**Figure 5:**
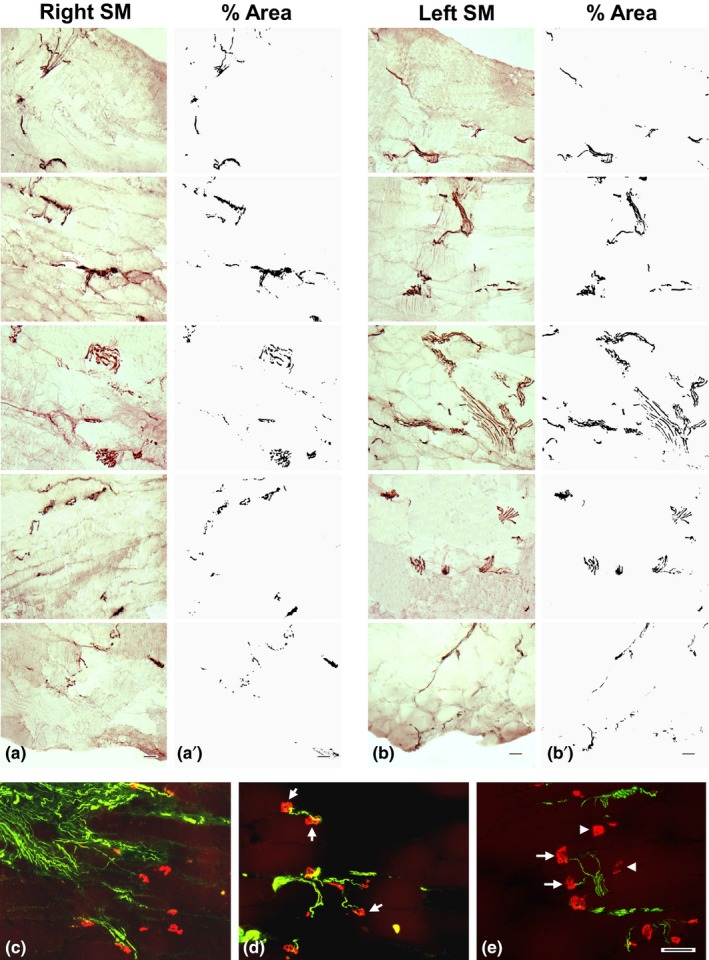
Images of the immunostained sections of the NMEG‐NMZ reinnervated and contralateral control SM muscles of a rat. (a–b) Images of sagittal sections of the right reinnervated (a) and left control (b) SM muscles. The sections were immunostained with antibody against neurofilament (NF) and photographed from ventral (top) to dorsal (bottom) surfaces of the muscle. Note that the nerve fascicles and axons (darkly stained threads and dots) in the right NMEG‐NMZ reinnervated SM are distributed throughout the thickness of the muscle. (a'–b') The stained sections in a and b were opened using ImageJ software and converted to 8‐bit (binary) images, color thresholded, and particle analyzed for nerve morphometry. The density of the axons was evaluated by estimating the number and area fraction of the NF‐positive axons within a section area (1.0 mm^2^). For this rat, as compared with the left control SM (mean axon count: 398; mean area: 2.92) the right SM exhibited very good muscle reinnervation as indicated by the mean axon count (283; 71% of the control) and the mean area (2.32; 80% of the control). Bar = 100 μm for a through b. (c–e) Images of sagittal sections immunostained with double fluorescence staining of the right reinnervated SM muscle of a rat, showing axon‐endplate connections. Regenerated axons (green) were detected with SMI‐31 monoclonal against neurofilaments, while motor endplates (MEPs; red) were labeled with α‐bungarotoxin. Note that the regenerated axons branched extensively into fields of MEPs in the NMZ of the target muscle (c). The majority of the MEPs in the treated muscle were reinnervated by regenerated axons (arrows in e), while some MEPs in the same muscle were unoccupied by regenerated axons (arrowheads in d and e). Bar = 100 μm for c through e

The muscle sections from NMEG‐NMZ reinnervated SM processed with NF staining exhibited abundance of NF‐ir axons. In the reinnervated SM, the regenerating axons from the implanted NMEG supplied the denervated NMZ and reached the most distal portion of the muscle, achieving almost full muscle reinnervation (Figure 5a–b). The mean number and area fraction of the labeled axons for each muscle in each animal is summarized in Table 2. On average, the regenerated axons in the NMEG‐NMZ reinnervated muscles were calculated to be 76.8% of the controls. No NF‐ir axons were identified in the stained sections of the denervated SM muscles (data not shown).

**Table 2:** Quantitative comparison of count and %area of neurofilament‐immunoreactive axons between right operated and left control sternomastoid (SM) muscles in rats (*n *= 13)

Animal no.	Right SM		Left SM		Ratio (R/L)	
	Count	%Area	Count	%Area	Count	%Area
1	765	0.366	980	0.681	0.781	0.537
2	652	0.494	854	0.544	0.763	0.908
3	726	0.925	996	1.081	0.729	0.856
4	769	0.475	1004	0.782	0.766	0.607
5	494	0.305	708	0.478	0.698	0.638
6	822	0.303	1065	0.463	0.772	0.654
7	857	0.544	1091	1.133	0.786	0.480
8	583	0.264	696	0.484	0.838	0.545
9	576	0.601	854	0.701	0.674	0.857
10	904	0.773	1096	0.844	0.825	0.916
11	766	0.457	871	0.493	0.879	0.927
12	289	1.073	381	0.968	0.759	1.108
13	283	2.320	398	2.915	0.711	0.796
Average	653	0.685	846	0.890	0.768	0.756

L, left; R, right.

The sagittal sections of the treated SM muscles immunostained with double fluorescence staining showed the innervated and noninnervated MEPs. In the SM muscles reinnervated with NMEG‐NMZ, the regenerating axons grew across the NMZ to innervate the denervated MEPs (Figure 5c–e). The majority of the denervated MEPs (80%) in the treated muscles were reinnervated by regenerating axons, while the remaining MEPs were unoccupied by axons. In addition, axonal sprouts and newly formed small MEPs were identified in the NME‐NMZ reinnervated muscles. In the stained sections from the denervated SM muscles, no axons were visualized and the denervated MEPs were characterized by reduced staining density, decreased number, and altered shape and size (data not shown).

## 4 Discussion

In this study, we have demonstrated that NMEG‐NMZ technique resulted in optimal recovery of muscle force (82% of the control). The reinnervated muscles exhibited good preservation of muscle mass (89% of the control) and myofiber morphology. In the treated muscles, the mean count and area of the axons reached up to 76.8% and 75.6% of the controls, respectively, and the majority (80%) of the denervated MEPs regained motor innervation. The optimal outcomes are attributed to the unique advantages of the NMEG‐NMZ technique. Specifically, the regenerating axons from the NMEG could rapidly reinnervate the denervated MEPs in the target muscle and form functional synapses.

A transferred NMEG could provide an abundant source of nerve terminals and MEPs for nerve regeneration and muscle reinnervation. NMEG has sufficient pedicle‐recipient muscle interfaces, which provide enough space for axonal regeneration. The axons could start to regenerate at multiple points in the implanted NMEG and grow across the pedicle‐recipient muscle interfaces to reach the recipient muscle fibers. The NMEG‐NMZ technique described here is based on the concept that denervated MEPs in the NMZ of the target muscle are preferential sites for reinnervation. One reason for the impaired target reinnervation could be degradation of MEPs during prolonged denervation. The NMEG‐NMZ procedure physically shortens regeneration distances and facilitates rapid MEP reinnervation to avoid irreversible loss of the denervated MEPs in the target muscle. The importance of nerve regeneration onto the sites of the original MEPs is highlighted by various animal experiments. Recent studies (Ma et al., 2011) have shown that poor motor recovery after peripheral nerve injury resulted from a failure of synapse reformation because of the delay in motor axons reaching their target. Barbour, Yee, Kahn, and Mackinnon (2012) emphasized that *“Functional motor recovery after peripheral nerve injury is predominantly determined by the time to motor endplate reinnervation and the absolute number of regenerated motor axons that reach target.”* The MEP is a highly specialized structure, optimized for the rapid transmission of information from the presynaptic nerve terminal to the postsynaptic muscle fiber. It serves to efficiently communicate the electrical impulse from the motor neuron to the skeletal muscle to signal contraction. MEP regions of mammalian muscle fibers are preferentially reinnervated as a consequence of some special property of the muscle fiber at this site. Studies have demonstrated that after nerve injury regenerating axons preferentially form synapses at original synaptic sites (Bennett, McLachlan, & Taylor, 1973; Covault, Cunningham, & Sanes, 1987; Gorio, Carmignoto, Finesso, Polato, & Nunzi, 1983; Sanes, Marshall, & McMahan, 1978). As reported, 30 days after nerve transection, all the regenerating nerve fibers grew into and innervated the regions of the original MEPs (Iwayama, 1969). The MEPs may exert an attraction on the regenerating axons. Synaptic basal lamina at the MEP contains molecules that direct the formation of synaptic specializations on regenerating axon terminals and myofibers (McMahan & Wallace, 1989). Some other chemotropic substance released from the MEPs may attract the regenerating axons in the vicinity. Using direct nerve implantation model, some investigators observed preferential reinnervation of the native MEPs in the target muscle by abundant regenerating axons and sprouts (Guth & Zalewski, 1963; Sakellarides, Sorbie, & James, 1972).

NMEG‐NMZ technique would have the potential for clinical application. It could be considered in selected clinical cases with extremity nerve injuries when no other repair options are possible. This method would be useful especially for treating laryngeal and facial paralysis. One of the most important problems involved in rehabilitation surgery of the recurrent laryngeal nerve (RLN) arises from the fact that this nerve carries fibers innervating antagonistic muscle groups (abductor and adductor muscles) which perform different functions during phonation, respiration, and swallowing. After RLN damage or nerve repair, some axons grow in a misdirected fashion. This leads to adductor axons innervating abductor muscles and vice versa*,* resulting in “synkinesis”, in which both opening (abductor) and closing (adductor) muscles activate in a dysfunctional way (Crumley, 2000). Synkinetic reinnervation is responsible for poor functional outcome as a result of simultaneous contraction of antagonistic muscles. To avoid synkinesis and functional failures, selective reinnervation of the glottis opener and of the glottis‐closing musculature is commonly performed. Up to date, ansa cervicalis is a commonly used candidate as a potential donor for selective reinnervation of the laryngeal adductor and abductor muscles (Crumley, 1991). The donor nerve can be directly implanted into a target muscle or prepared as a nerve‐muscle pedicle (NMP) to reinnervate the paralyzed muscle (Tucker, 1989). The NMP method involves transferring a donor nerve branch with a small piece of muscle tissue (a 2‐ to 3‐mm cube) from a cervical strap muscle to a paralyzed laryngeal muscle. Surgical methods for reanimation of the paralyzed face include cross‐facial nerve graft, nerve transfers, and free muscle transplantation (Hoffman, 1992; Terzis & Konofaos, 2008). The NMP transfer or direct nerve implantation has been also used for the selective reinnervation of paralyzed facial muscles to avoid synkinesis (Goding et al., 1989; Hall, Trachy, & Cummings, 1988). Although some authors (Tucker, 1989) reported good results, others reported poor muscle reinnervation and functional recovery (Fata, Malmgren, Gacek, Dum, & Woo, 1987; Rice & Burstein, 1983) after NMP transfer. There are several factors leading to poor outcome. First, a NMP is designed at the point where a nerve branch enters the muscle without consideration of the MEPs and nerve terminals. Second, the pedicle is too small to contain donor nerve terminals and MEPs. Finally, the pedicle may contain only a nerve stump, serving as direct nerve implantation as both methods resulted in similar outcomes (Goding et al., 1989; Hall et al., 1988). The results from this study suggest that NMEG‐NMZ technique could be an option for the treatment of laryngeal and facial paralysis.

Although our experiments showed the potential of NNMEG‐NMZ in immediate muscle reinnervation, this study also has some limitations. For example, postoperative evaluations were performed at the end of 3 months after surgery. Three‐month recovery period may be not enough to provide a complete picture of what occurs after NMEG‐NMZ. Further studies are needed to determine morphological and functional alterations at different time points after NMEG‐NMZ reinnervation. This information would be helpful for better understanding the time‐related changes of muscle reinnervation and functional recovery. In addition, it remains unknown if the NMEG‐NMZ has the potential for delayed reinnervation that is not uncommon in clinical practice. One of our ongoing studies is to determine the efficacy of NMEG‐NMZ for chronic muscle denervation. For this purpose, it is important to know the decreasing rate of the endplates in the completely denervated muscle and to determine the time point when all the endplates cannot be detected.

In conclusion, this study showed that the NMZ of the target muscle is the best site in the target muscle for NMEG implantation. NMEG‐NMZ procedure could shorten axon regeneration distances and facilitate rapid endplate reinnervation, thereby avoiding muscle atrophy and contributing to optimal functional recovery. Our results have demonstrated that NMEG‐NMZ technique resulted in extensive axonal regeneration, MEP reinnervation, and optimal functional recovery. This method can be used for treatment of laryngeal and facial paralysis. Further technical improvements and studies to identify the NMZ in laryngeal musculature will be required before this technique can be applied in a clinically meaningful fashion. In addition, it could be one alternative to reinnervate other denervated muscles when no other repair options are possible. Although NMEG‐NMZ technique has treatment potential in immediate muscle reinnervation, further work is warranted to determine its effectiveness for delayed muscle reinnervation.

## Conflict of Interest

None declared.

## References

[brb3668-bib-0001] Al‐Qattan, M. M. (2001). Terminolateral neurorrhaphy: Review of experimental and clinical studies. Journal of Reconstructive Microsurgery, 17, 99–108. 11310757 10.1055/s-2001-12698

[brb3668-bib-0002] Barbour, J. , Yee, A. , Kahn, L. C. , & Mackinnon, S. E. (2012). Supercharged end‐to‐side anterior interosseous to ulnar motor nerve transfer for intrinsic musculature reinnervation. The Journal of Hand Surgery, 37, 2150–2159. 23021177 10.1016/j.jhsa.2012.07.022

[brb3668-bib-0003] Bennett, M. R. , McLachlan, E. M. , & Taylor, R. S. (1973). The formation of synapses in reinnervated mammalian striated muscle. Journal of Physiology, 233, 481–500. 4127828 10.1113/jphysiol.1973.sp010319 PMC1350588

[brb3668-bib-0004] Brininger, T. L. , Antczak, A. , & Breland, H. L. (2008). Upper extremity injuries in the U.S. military during peacetime years and wartime years. Journal of Hand Therapy, 21, 115–122. 18436132 10.1197/j.jht.2007.10.010

[brb3668-bib-0005] Brunelli, G. A. , & Brunelli, L. M. (1980). Direct neurotization of severely damaged denervated muscles. International Surgery, 65, 529–531. 7203873

[brb3668-bib-0006] Brunelli, G. A. , & Brunelli, G. R. (1993). Direct muscle neurotization. Reconstructive Microsurgery, 9, 81–90. 10.1055/s-2007-1006656 8468705

[brb3668-bib-0007] Covault, J. , Cunningham, J. M. , & Sanes, J. R. (1987). Neurite outgrowth on cryostat sections of innervated and denervated skeletal muscle. Journal of Cell Biology, 105, 2479–2488. 3693390 10.1083/jcb.105.6.2479 PMC2114719

[brb3668-bib-0008] Crumley, R. L. (1991). Update: Ansa cervicalis to recurrent laryngeal nerve anastomosis for unilateral laryngeal paralysis. Laryngoscope, 101, 384–388. 1895854 10.1002/lary.1991.101.4.384

[brb3668-bib-0009] Crumley, R. L. (2000). Laryngeal synkinesis revisited. The Annals of Otology, Rhinology, and Laryngology, 109, 365–371. 10.1177/000348940010900405 10778890

[brb3668-bib-0010] De Sa, J. M. , Mazzer, N. , Barbieri, C. H. , & Barriera, A. A. (2004). The end‐to‐side peripheral nerve repair. Functional and morphometric study using the peroneal nerve of rats. Journal of Neuroscience Methods, 136, 45–53. 15126044 10.1016/j.jneumeth.2003.12.018

[brb3668-bib-0011] Eser, F. , Aktekin, L. A. , Bodur, H. , & Atan, C. (2009). Etiological factors of traumatic peripheral nerve injuries. Neurol India, 57, 434–437. 19770544 10.4103/0028-3886.55614

[brb3668-bib-0012] Fata, J. J. , Malmgren, L. T. , Gacek, R. R. , Dum, R. , & Woo, P. (1987). Histochemical study of posterior cricoarytenoid muscle reinnervation by a nerve‐muscle pedicle in the cat. The Annals of Otology, Rhinology, and Laryngology, 96, 479–487. 10.1177/000348948709600501 3674642

[brb3668-bib-0013] Goding, G. S. Jr , Cummings, C. W. , & Bright, D. A. (1989). Extension of neuromuscular pedicles and direct nerve implants in the rabbit. Archives of Otolaryngology ‐ Head and Neck Surgery, 115, 217–223. 2914094 10.1001/archotol.1989.01860260091021

[brb3668-bib-0014] Gorio, A. , Carmignoto, G. , Finesso, M. , Polato, P. , & Nunzi, M. G. (1983). Muscle reinnervation. II. Sprouting, synapse formation, and repression. Neuroscience, 8, 403–416. 6856082 10.1016/0306-4522(83)90188-4

[brb3668-bib-0015] Green, D. C. , Berke, G. S. , & Graves, M. C. (1991). A functional evaluation of ansa cervicalis nerve transfer for unilateral vocal cord paralysis: Future directions for laryngeal reinnervation. Otolaryngology ‐ Head and Neck Surgery, 104, 453–466. 1903856 10.1177/019459989110400406

[brb3668-bib-0016] Grumbles, R. M. , Sesodia, S. , Wood, P. M. , & Thomas, C. K. (2009). Neurotrophic factors improve motoneuron survival and function of muscle reinnervated by embryonic neurons. Journal of Neuropathology and Experimental Neurology, 68, 736–746. 19535998 10.1097/NEN.0b013e3181a9360f PMC2727878

[brb3668-bib-0017] Guth, L. , & Zalewski, A. A. (1963). Disposition of cholinesterase following implantation of nerve into innervated and denervated muscle. Experimental Neurology, 7, 316–326. 13951420 10.1016/0014-4886(63)90078-5

[brb3668-bib-0018] Hall, S. J. , Trachy, R. E. , & Cummings, C. W. (1988). Facial muscle reinnervation: A comparison of neuromuscular pedicle with direct nerve implant. The Annals of Otology, Rhinology, and Laryngology, 97, 229–233. 10.1177/000348948809700303 3377390

[brb3668-bib-0019] Hoffman, W. Y. (1992). Reanimation of the paralyzed face. Otolaryngologic Clinics of North America, 25, 649–667. 1625868

[brb3668-bib-0020] Ichihara, S. , Inada, Y. , & Nakamura, T. (2008). Artificial nerve tubes and their application for repair of peripheral nerve injury: An update of current concepts. Injury, 39(Suppl 4), 29–39. 18804584 10.1016/j.injury.2008.08.029

[brb3668-bib-0021] Iwayama, T. (1969). Relation of regenerating nerve terminals to original endplates. Nature, 224, 81–82. 5822915 10.1038/224081a0

[brb3668-bib-0022] Kelsey, J. , Praemer, A. , Nelson, L. , Felberg, A. , & Rice, D. (1997). Upper extremity disorders: Frequency, impact, and cost. London: Churchhill‐Livingstone.

[brb3668-bib-0023] Kretschmer, T. , Antoniadis, G. , Braun, V. , Rath, S. A. , & Richter, H. P. (2001). Evaluation of iatrogenic lesions in 722 surgically treated cases of peripheral nerve trauma. Journal of Neurosurgery, 94, 905–912. 11409518 10.3171/jns.2001.94.6.0905

[brb3668-bib-0024] Lee, S. K. , & Wolfe, S. W. (2000). Peripheral nerve injury and repair. Journal of American Academy of Orthopaedic Surgeons, 8, 243–252. 10.5435/00124635-200007000-00005 10951113

[brb3668-bib-0025] Lee, S. K. , & Wolfe, S. W. (2012). Nerve transfers for the upper extremity: New horizons in nerve reconstruction. Journal of American Academy of Orthopaedic Surgeons, 20, 506–517. 10.5435/JAAOS-20-08-506 22855853

[brb3668-bib-0026] Ma, C. H. , Omura, T. , Cobos, E. J. , Latremoliere, A. , Ghasemlou, N. , Brenner, G. J. , … Woolf, C. J. (2011). Accelerating axonal growth promotes motor recovery after peripheral nerve injury in mice. Journal of Clinical Investigation, 121, 4332–4347. 21965333 10.1172/JCI58675 PMC3223863

[brb3668-bib-0027] Marie, J. P. , Lerosey, Y. , Dehesdin, D. , Jin, O. , Tadie, M. , & Andrieu‐Guitrancourt, J. (1999). Experimental reinnervation of a strap muscle with a few roots of the phrenic nerve in rabbits. The Annals of Otology, Rhinology, and Laryngology, 108, 1004–1011. 10.1177/000348949910801013 10526857

[brb3668-bib-0028] McAllister, R. M. , Gilbert, S. E. , Calder, J. S. , & Smith, P. J. (1996). The epidemiology and management of upper limb peripheral nerve injuries in modern practice. The Journal of Hand Surgery, 21, 4–13. 8676027 10.1016/s0266-7681(96)80004-0

[brb3668-bib-0029] McMahan, U. J. , & Wallace, B. G. (1989). Molecules in basal lamina that direct the formation of synaptic specializations at neuromuscular junctions. Developmental Neuroscience, 11, 227–247. 2676454 10.1159/000111903

[brb3668-bib-0030] de Medinaceli, L. , Prayon, M. , & Merle, M. (1993). Percentage of nerve injuries in which primary repair can be achieved by end‐to‐end approximation: Review of 2,181 nerve lesions. Microsurgery, 14, 244–246. 8412633 10.1002/micr.1920140406

[brb3668-bib-0031] Meikle, D. , Trachy, R. E. , & Cummings, C. W. (1987). Reinnervation of skeletal muscle: A comparison of nerve implantation with neuromuscular pedicle transfer in an animal model. The Annals of Otology, Rhinology, and Laryngology, 96, 152–157. 10.1177/000348948709600203 3551740

[brb3668-bib-0032] Merle, M. , Dellon, A. L. , Campbell, J. N. , & Chang, P. S. (1989). Complications from silicon‐polymer intubulation of nerves. Microsurgery, 10, 130–133. 2770512 10.1002/micr.1920100213

[brb3668-bib-0033] van der Meulen, J. H. , Urbanchek, M. G. , Cederna, P. S. , Eguchi, T. , & Kuzon, W. M. Jr (2003). Denervated muscle fibers explain the deficit in specific force following reinnervation of the rat extensor digitorum longus muscle. Plastic and Reconstructive Surgery, 112, 1336–1346. 14504517 10.1097/01.PRS.0000081464.98718.E3

[brb3668-bib-0034] Moneim, M. , & Omer, G. (1998). Clinical outcome following acute nerve repair In Omeg G., Spinner M., & Van Beek A. (Eds.), Management of peripheral nerve problems (pp. 414–419). Philadelphia, PA: Saunders.

[brb3668-bib-0035] Mu, L. , Chen, J. , Sobotka, S. , Nyirenda, T. , Benson, B. , Gupta, F. , … Beach, T. (2015). Alpha‐synuclein pathology in sensory nerve terminals of the upper aerodigestive tract of Parkinson's disease patients. Dysphagia, 30, 404–417. 26041249 10.1007/s00455-015-9612-7 PMC4503503

[brb3668-bib-0036] Mu, L. , & Sanders, I. (2010). Sihler's whole mount nerve staining technique: A review. Biotechnic and Histochemistry, 85, 19–42. 19572223 10.1080/10520290903048384 PMC2822894

[brb3668-bib-0037] Mu, L. , Sobotka, S. , & Su, H. (2011). Nerve‐muscle‐endplate band grafting: A new technique for muscle reinnervation. Neurosurgery, 69(Suppl. 2), 208–224. 10.1227/NEU.0b013e31822ed596 PMC3204339 21796004

[brb3668-bib-0038] Myckatyn, T. M. , & Mackinnon, S. E. (2004). A review of research endeavors to optimize peripheral nerve reconstruction. Neurological Research, 26, 124–138. 15072631 10.1179/016164104225013743

[brb3668-bib-0039] Noble, J. , Munro, C. A. , Prasad, V. S. , & Midha, R. (1998). Analysis of upper and lower extremity peripheral nerve injuries in a population of patients with multiple injuries. Journal of Trauma, 45, 116–122. 9680023 10.1097/00005373-199807000-00025

[brb3668-bib-0040] Rice, D. H. , & Burstein, F. D. (1983). Restoration of physiologic vocal fold abduction with the ansa cervicalis nerve. Archives of Otolaryngology, 109, 480–481. 6344850 10.1001/archotol.1983.00800210056014

[brb3668-bib-0041] Sakellarides, H. T. , Sorbie, C. , & James, L. (1972). Reinnervation of denervated muscles by nerve transplantation. Clinical Orthopaedics and Related Research, 83, 195–201. 5014813

[brb3668-bib-0042] Sanapanich, K. , Morrison, W. A. , & Messina, A. (2002). Physiologic and morphologic aspects of nerve regeneration after end‐to‐end or end‐to‐side coaptation in a rat model of brachial plexus injury. The Journal of Hand Surgery, 27, 133–142. 11810627 10.1053/jhsu.2002.30370

[brb3668-bib-0043] Sanes, J. R. , Marshall, L. M. , & McMahan, U. J. (1978). Reinnervation of muscle fiber basal lamina after removal of myofibers. Differentiation of regenerating axons at original synaptic sites. Journal of Cell Biology, 78, 176–198. 307554 10.1083/jcb.78.1.176 PMC2110176

[brb3668-bib-0044] Siemionow, M. , & Brzezicki, G. (2009). Chapter 8: Current techniques and concepts in peripheral nerve repair. International Review of Neurobiology, 87, 141–172. 19682637 10.1016/S0074-7742(09)87008-6

[brb3668-bib-0045] Sobotka, S. , & Mu, L. (2010). Characteristics of tetanic force produced by the sternomastoid muscle of the rat. Journal of Biomedicine & Biotechnology, 2010, 194984. 20508813 10.1155/2010/194984 PMC2875700

[brb3668-bib-0046] Sobotka, S. , & Mu, L. (2011). Force characteristics of the rat sternomastoid muscle reinnervated with end‐to‐end nerve repair. Journal of Biomedicine & Biotechnology, 2011, 173471. 22203781 10.1155/2011/173471 PMC3238804

[brb3668-bib-0047] Sobotka, S. , & Mu, L. (2013a). Force recovery and axonal regeneration of the sternomastoid muscle reinnervated with the end‐to‐end nerve anastomosis. Journal of Surgical Research, 182, e51–e59. 23207170 10.1016/j.jss.2012.11.003 PMC3593814

[brb3668-bib-0048] Sobotka, S. , & Mu, L. (2013b). Comparison of muscle force after immediate and delayed reinnervation using nerve‐muscle‐endplate band grafting. Journal of Surgical Research, 179, e117–e126. 22480827 10.1016/j.jss.2012.02.055 PMC3393842

[brb3668-bib-0049] Sobotka, S. , & Mu, L. (2015). Muscle reinnervation with nerve‐muscle‐endplate band grafting technique: Correlation between force recovery and axonal regeneration. Journal of Surgical Research, 195, 144–151. 25661741 10.1016/j.jss.2015.01.008 PMC4385406

[brb3668-bib-0050] Stanec, S. , & Stanec, Z. (1998). Reconstruction of upper‐extremity peripheral‐nerve injuries with ePTFE conduits. Journal of Reconstructive Microsurgery, 14, 227–232. 9618088 10.1055/s-2007-1000173

[brb3668-bib-0051] Terzis, J. K. , & Konofaos, P. (2008). Nerve transfers in facial palsy. Facial Plastic Surgery, 24, 177–193. 18470829 10.1055/s-2008-1075833

[brb3668-bib-0052] Tucker, H. M. (1989). Long‐term results of nerve‐muscle pedicle reinnervation for laryngeal paralysis. The Annals of Otology, Rhinology, and Laryngology, 98, 674–676. 10.1177/000348948909800903 2782800

[brb3668-bib-0053] Wiberg, M. , & Terenghi, G. (2003). Will it be possible to produce peripheral nerves? Surgical Technology International, 11, 303–310. 12931315

[brb3668-bib-0054] Wong, B. J. , & Crumley, R. L. (1995). Nerve wound healing. An overview. Otolaryngologic Clinics of North America, 28, 881–895. 8559578

[brb3668-bib-0055] Yoshimura, K. , Asato, H. , Jejurikar, S. S. , Cederna, P. S. , Urbanchek, M. G. , & Kuzon, W. M. Jr (2002). The effect of two episodes of denervation and reinnervation on skeletal muscle contractile function. Plastic and Reconstructive Surgery, 109, 212–219. 11786814 10.1097/00006534-200201000-00032

[brb3668-bib-0056] Zhang, X. , Mu, L. , Su, H. , & Sobotka, S. (2011). Locations of the motor endplate band and motoneurons innervating the sternomastoid muscle in the rat. Anatomical Record (Hoboken), 294, 295–304. 10.1002/ar.21312 PMC3040007 21235005

